# New Frontiers in Electrocardiography, Cardiac Arrhythmias, and Arrhythmogenic Disorders

**DOI:** 10.3390/jcm13072047

**Published:** 2024-04-01

**Authors:** Rafał Król, Michał Karnaś, Michał Ziobro, Jacek Bednarek, Georgios Kollias, Christian Sohns, Paweł T. Matusik

**Affiliations:** 1 Department of Electrocardiology, St. John Paul II Hospital, Prądnicka 80, 31-202 Kraków, Poland; 2 Faculty of Medicine, Jagiellonian University Medical College, Św. Anny 12, 31-008 Kraków, Poland; 3 Ordensklinikum Linz Elisabethinen, Fadingerstraße 1, 4020 Linz, Austria; 4 Clinic for Electrophysiology, Herz- und Diabeteszentrum NRW, Georgstr. 11, 32545 Bad Oeynhausen, Germany; 5 Department of Electrocardiology, Institute of Cardiology, Faculty of Medicine, Jagiellonian University Medical College, Prądnicka 80, 31-202 Kraków, Poland

In recent decades, diagnosing, risk-stratifying, and treating patients with primary electrical diseases, as well as heart rhythm disorders, have improved substantially. Moreover, new clinical classification of rare cardiac arrhythmogenic and conduction disorders and rare arrhythmias has been proposed to facilitate research and simplify differential diagnostics [1]. Significant progress has been made in assessing the genetic background of patients after sudden cardiac arrest [2], and new frontiers have also been reached in the field of stratifying cardiovascular risk and predicting sudden cardiac death. These advances concern the use of novel biomarkers in combination with clinical data [3,4]. Artificial intelligence may also be useful in addressing unmet needs in improving the prediction of sudden cardiac arrest [5]. In current clinical practice, the use of conduction system pacing and subcutaneous implantable cardioverter–defibrillator therapy is increasingly common in patients who require cardiac pacing or sudden cardiac death prevention with the use of implantable cardioverter–defibrillators [6,7]. Importantly, patients at high risk of pacemaker pocket infections or with a lack of upper-extremity venous access may benefit from leadless pacemakers, which may preserve (at least to some extent) atrioventricular synchrony, such as a novel dual-chamber leadless pacemaker system [8,9]. Moreover, there is increasing clinical evidence on the safe extraction of leadless pacemakers with a dwelling time of over 12 months [10].

Important new technologies have been introduced in the field of ablation procedures since cardiac ablations were previously performed with the delivery of direct current shocks to an electrode catheter [11]. Due to the severe complications involved, this was replaced by radiofrequency ablation, which is currently widely used in electrophysiology laboratories. The use of steerable sheaths, contact force sensing (the contact force is positively correlated with the lesion size, steam pops, and thrombus formation), and irrigated tip catheters (larger lesions creation, a lower risk of thrombus and char formation) for ablation has brought about improvements in the field of radiofrequency ablation [12,13,14,15]. The catheters may be visualized using fluoroscopy or three-dimensional electroanatomical mapping techniques, limiting or completely omitting the use of X-rays (zero X-ray ablation) and increasing the precision of ablating the arrhythmogenic substrate. Another ablation method is cryoablation, which may be especially valuable in pulmonary vein isolation and in patients with perinodal accessory pathways due to the low risk of persistent iatrogenic atrioventricular block [16]. Moreover, the use of pulse field ablation also holds great promise, which is a novel ablation modality utilizing non-thermal energy and causing irreversible electroporation, leading to cardiac cell death (other cells are less prone to these changes) [17,18]. Among patients who experience recurrent ventricular tachycardias after catheter ablation despite optimal pharmacotherapy or among those who have contraindications to catheter ablation, stereotactic arrhythmia radioablation is a potentially valuable treatment option [19]. 

In the Special Issue “New Frontiers in Electrocardiography, Cardiac Arrhythmias, and Arrhythmogenic Disorders” of the Journal of Clinical Medicine, readers will find 22 papers (summarized in Table 1) written by authors from around the world (Figure 1). These papers concern a variety of topics in the field of electrocardiology [20,21,22].

In the rapidly growing field of electrocardiology, further in-depth analyses of pathogenesis and clinical management, along with important aspects of telehealth, which is promising for the improvement of care [23], are needed. These should include detailed descriptions of case reports and systematic reviews, as seen in other fields of medicine [24,25]. Despite progress, there are gaps in our knowledge of and the clinical care of patients, including those with heart failure, who may benefit from the placement of implantable cardiovascular electronic devices (and their remote monitoring), catheter ablation, or optimized medical therapy [26,27]. Thus, we look forward to reading, reviewing, and/or editing new papers submitted to the Journal of Clinical Medicine, including the new Special Issue “Further Advances in Electrocardiography, Cardiac Arrhythmias, and Arrhythmogenic Disorders”.

## Figures and Tables

**Figure 1:**
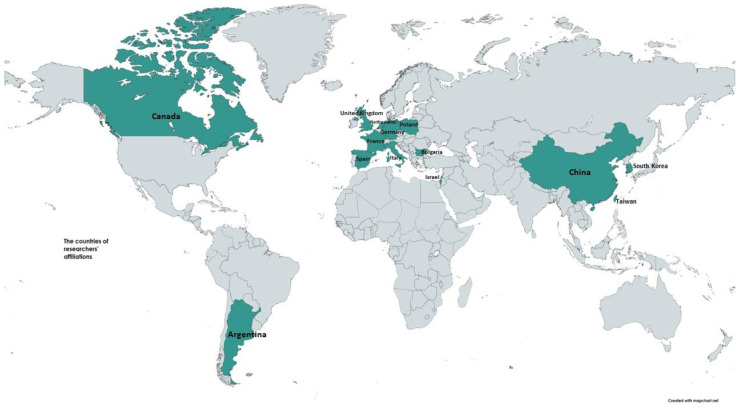
Locations of affiliations of the authors contributing to the Special Issue “New Frontiers in Electrocardiography, Cardiac Arrhythmias, and Arrhythmogenic Disorders”.

**Table 1:** Summary of the papers published in the Special Issue “New Frontiers in Electrocardiography, Cardiac Arrhythmias, and Arrhythmogenic Disorders”.

Contribution Number	Reference	Type of Publication	Number of Authors/Affiliations	Locations of Authors’ Affiliations
1	Bijak, P.; et al. Fever-Induced Brugada Sign: Clue for Clinical Management with Non-Negligible Risk of Sudden Cardiac Death. J. Clin. Med. 2023, 12.	Editorial	6/7	Poland, Bulgaria, Israel, Italy, Germany
2	Bun, S.-S.; et al. Prevalence and Clinical Characteristics of Patients with Torsades de Pointes Complicating Acquired Atrioventricular Block. J. Clin. Med. 2023, 12, 1067.	Article	7/1	France
3	Lisicka, M.; et al. Heart Rate Variability Impairment Is Associated with Right Ventricular Overload and Early Mortality Risk in Patients with Acute Pulmonary Embolism. J. Clin. Med. 2023, 12, 753.	Article	6/2	Poland
4	Buś, S.; et al. Statistical and Diagnostic Properties of pRRx Parameters in Atrial Fibrillation Detection. J. Clin. Med. 2022, 11, 5702.	Article	3/2	Poland
5	Buś, S.; et al. Using Minimum Redundancy Maximum Relevance Algorithm to Select Minimal Sets of Heart Rate Variability Parameters for Atrial Fibrillation Detection. J. Clin. Med. 2022, 11, 4004.	Article	3/2	Poland
6	Giovanardi, P.; et al. Combined Effects of Age and Comorbidities on Electrocardiographic Parameters in a Large Non-Selected Population. J. Clin. Med. 2022, 11, 3737.	Article	6/7	Italy
7	Matusik, P.S.; et al. Clinical Data, Chest Radiograph and Electrocardiography in the Screening for Left Ventricular Hypertrophy: The CAR_2_E_2_ Score. J. Clin. Med. 2022, 11, 3585.	Article	5/5	Poland
8	Tymińska, A.; et al. Fifteen-Year Differences in Indications for Cardiac Resynchronization Therapy in Inter-national Guidelines—Insights from the Heart Failure Registries of the European Society of Cardiology. J. Clin. Med. 2022, 11, 3236.	Article	11/5	Poland, Spain, Italy, France
9	Li, G.-Y.; et al. Sinus Node Dysfunction after Successful Atrial Flutter Ablation during Follow-Up: Clinical Characteristics and Predictors. J. Clin. Med. 2022, 11, 3212.	Article	16/3	Taiwan
10	Okólska, M.; et al. Prevalence of Arrhythmia in Adults after Fontan Operation. J. Clin. Med. 2022, 11, 1968.	Article	9/7	Poland
11	Kowalik, R.; et al. In-Hospital and One-Year Outcomes of Patients after Early and Late Resuscitated Cardiac Arrest Complicating Acute Myocardial Infarction—Data from a Nationwide Database. J. Clin. Med. 2022, 11, 609.	Article	10/4	Poland
12	Karkowski, G.; et al. Contact Force-Sensing versus Standard Catheters in Non-Fluoroscopic Radiofrequency Catheter Ablation of Idiopathic Outflow Tract Ventricular Arrhythmias. J. Clin. Med. 2022, 11, 593.	Article	7/5	Poland
13	Bun, S.-S.; et al. Prevalence and Clinical Characteristics of Patients with Pause-Dependent Atrioventricular Block. J. Clin. Med. 2022, 11, 449.	Article	9/2	France
14	Ozierański, K.; et al. Sex Differences in Incidence, Clinical Characteristics and Outcomes in Children and Young Adults Hospitalized for Clinically Suspected Myocarditis in the Last Ten Years—Data from the MYO-PL Nationwide Database. J. Clin. Med. 2021, 10, 5502.	Article	8/3	Poland
15	Tsai, C.-F.; et al. Long-Term Prognosis of Febrile Individuals with Right Precordial Coved-Type ST-Segment Elevation Brugada Pattern: A 10-Year Prospective Follow-Up Study. J. Clin. Med. 2021, 10, 4997.	Article	4/3	Taiwan
16	Ozierański, K.; et al. Occurrence, Trends, Management and Outcomes of Patients Hospitalized with Clinically Suspected Myocarditis—Ten-Year Perspectives from the MYO-PL Nationwide Database. J. Clin. Med. 2021, 10, 4672.	Article	7/2	Poland
17	Okólska, M.; et al. Heart Rate Variability and Its Associations with Organ Complications in Adults after Fontan Operation. J. Clin. Med. 2021, 10, 4492.	Article	7/5	Poland
18	Wałek, P.; et al. Echocardiographic Evaluation of Atrial Remodelling for the Prognosis of Maintaining Sinus Rhythm after Electrical Cardioversion in Patients with Atrial Fibrillation. J. Clin. Med. 2023, 12, 5158.	Review	4/3	Poland
19	Aziz, H.M.; et al. Pathogenesis and Management of Brugada Syndrome: Recent Advances and Protocol for Umbrella Reviews of Meta-Analyses in Major Arrhythmic Events Risk Stratification. J. Clin. Med. 2022, 11, 1912.	Review	8/11	Poland, Argentina, Korea, China, the United Kingdom
20	He, M.; et al. Caveolin-3 and Arrhythmias: Insights into the Molecular Mechanisms. J. Clin. Med. 2022, 11, 1595.	Review	5/1	China
21	Ahmed, M.; et al. Rhythm vs. Rate Control in Patients with Postoperative Atrial Fibrillation after Cardiac Surgery: A Systematic Review and Meta-Analysis. J. Clin. Med. 2023, 12, 4534.	Systematic review	15/4	Canada
22	Oliva, A.; et al. Structural Heart Alterations in Brugada Syndrome: Is it Really a Channelopathy? A Systematic Review. J. Clin. Med. 2022, 11, 4406.	Systematic review	21/11	Spain, Italy, The Netherlands
